# Topological Phase Transition in Single Crystals of (Cd_1−x_Zn_x_)_3_As_2_

**DOI:** 10.1038/s41598-017-03559-2

**Published:** 2017-06-09

**Authors:** Hong Lu, Xiao Zhang, Yi Bian, Shuang Jia

**Affiliations:** 1 ICQM, School of Physics, Peking University, Beijing, 100871 China; 2 Collaborative Innovation Center of Quantum Matter, Beijing, 100871 China

## Abstract

Single crystals of (Cd_1−x_Zn_x_)_3_As_2_ were synthesized from high-temperature solutions and characterized in terms of their structural and electrical properties. Based on the measurements of resistivity and Hall signals, we revealed a chemical-doping-controlled transition from a three-dimensional Dirac semimetal to a semiconductor with a critical point x_c_ ~ 0.38. We observed structural transitions from a body-center tetragonal phase to a primitive tetragonal phase then back to a body-center tetragonal phase in the solid solutions as well, which are irrelevant to the topological phase transition. This continuously tunable system controlled by chemical doping provides a platform for investigating the topological quantum phase transition of three-dimensional Dirac electrons.

## Introduction

Cadmium and zinc pnictides (Cd_3_As_2_, Zn_3_As_2_, Cd_3_P_2_ and Zn_3_P_2_) belong to a group of ${{\rm{A}}}_{{\rm{3}}}^{{\rm{II}}}{{\rm{B}}}_{{\rm{2}}}^{{\rm{V}}}$ semiconductors and semimetals, which have been well known for their potential applications in highly efficient solar cells and optoelectronic devices^1–4^. These four compounds crystallize at various temperatures in several closely related structures, which can be viewed as the different arrangements of a distorted antifluorite structure^5–12^.

The electrical properties of these four compounds are distinct in several aspects. Zn_3_As_2_, Zn_3_P_2_ and Cd_3_P_2_ are semiconductors with low carrier mobility and the direct band gaps being 1.0 eV, 1.5 eV and 0.5 eV respectively^13–15^. Both Zn_3_P_2_ and Zn_3_As_2_ are p-type, while Cd_3_P_2_ and Cd_3_As_2_ are n-type^13–15^. On the other hand, previous studies on the optical properties of Cd_3_As_2_ suggested that it was a semiconductor with a narrow band gap around 0.1 eV^13^. The mobility for Cd_3_As_2_ was reported as high as 1.5 × 10^4^ cm^2^/Vs at room temperature^13^. For comparing, the hole mobility for Zn_3_As_2_ is only 10 cm^2^/Vs at room temperature^13^. Cd_3_As_2_ was believed to manifest an inverted band structure due to the spin-orbital coupling (SOC)^16, 17^ while the other three had normal band structures.

Recent studies on Cd_3_As_2_ have revealed the topological aspect of its electrical properties^18–26^. Band structure calculation predicted that Cd_3_As_2_ was a three-dimensional (3D) Dirac semimetal with the band inversion^18^. The energy dispersion of the Dirac electron is protected by the rotational symmetry along the crystallographic **c** axis in the tetragonal unit cell. The 3D Dirac cones of Cd_3_As_2_ have been observed in angle-resolved photoemission spectroscopy (ARPES)^19–21^. The Dirac-like band dispersion and the inverted band ordering was probed by the Landau level spectroscopy and quasiparticle interference in scanning tunneling microscopy (STM)^22^. Based on the electrical transport measurements, two experimental groups found an ultrahigh mobility of the Dirac electrons^23, 24^. A strongly sample-dependent, large linear magnetoresistance (MR) was observed in Cd_3_As_2_ at low temperatures^23^. The nonsaturating linear MR in n-type Cd_3_As_2_ up to 65 T was believed to result from its mobility fluctuations^26^. The anisotropic Fermi surface with two ellipsoids of Dirac electrons along the **c** axis was revealed from the angular dependent measurements of SdH oscillations^24^.

Noticing the opposite band orderings in Cd_3_As_2_ and the other three members in the family, we expect a band inversion transition in pseudo-binary compounds of (Cd_1−x_Zn_x_)_3_As_2_ and Cd_3_(As_1−x_P_x_)_2_. A band inversion transition due to the change of the SOC strength has been observed in solid solutions of semiconductors and semimetals such as Hg_1−x_Cd_x_Te and Pb_1−x_Sn_x_Se^27, 28^. Recent studies on the solid solutions of TlBiSe_2−x_S_x_ and Bi_2−x_In_x_Se_3_ have confirmed the existence of a quantum phase transition tuned by chemical doping from topological insulators to trivial band insulators^29, 30^. A topological phase transition from a 3D Dirac semimetal to a trivial semiconductor was predicted in Na_3_Bi_1−x_Sb_x_ and Cd_3_(As_1−x_P_x_)_2_ by first-principle calculations as well^31^.

The changes of the structures and physical properties of polycrystalline (Cd_1−x_Zn_x_)_3_As_2_ and Cd_3_(As_1−x_P_x_)_2_ were studied before^32–36^. (Cd_1−x_Zn_x_)_3_As_2_ crystallize in a primitive tetragonal structure^32^. The majorities undergo a crossover from n- to p-type when x increases in (Cd_1−x_Zn_x_)_3_As_2_^33, 34^, while the band gap increases linearly with the proportion of Zn, according to the magneto-optical measurements^35^. However the change of the topological properties of the electronic structure has not been addressed.

In this study, we report the single-crystalline (Cd_1−x_Zn_x_)_3_As_2_ obtained from high-temperature solution growth. Powder X-ray Diffraction (XRD) measurements revealed structural transitions from a body-center tetragonal phase for Cd_3_As_2_, to a primitive tetragonal phase for 0.07 ≤ x < 0.52, and then back to a body-center tetragonal phase for x > 0.52. The electrical resistivity and Hall measurements revealed a metal-insulator transition at a critical point x_c_ ~ 0.38. The analysis of the MR demonstrated a transition from a 3D Dirac semimetal to a trivial direct-gap semiconductor via the modulation of the SOC strength.

## Results

**Figure 1:**
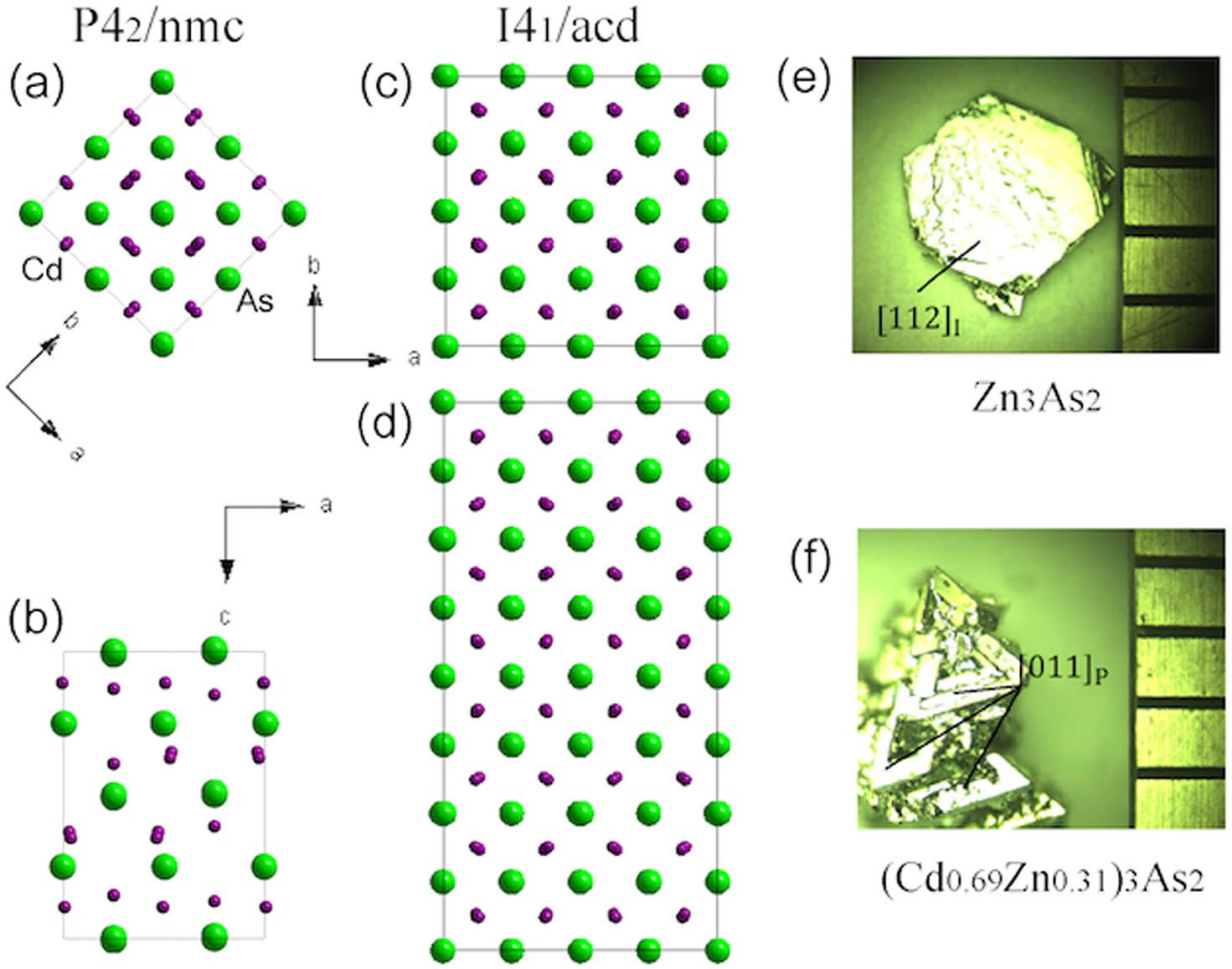
Panel (a–d): Unit cells of *α*″−Cd_3_As_2_ and *α*−Cd_3_As_2_ viewed along **c** and **b** axes respectively. Panel (e,f): Single crystals of Zn_3_As_2_ and (Cd_0.69_Zn_0.31_)_3_As_2_ show different morphologies (see more details in the text). The scale on right is 1 mm.

The low temperature phases of Cd_3_As_2_ and Zn_3_As_2_ were reported as *α*″ (P4_2_/nmc), *α*′ (P4_2_/nbc) and *α* (I4_1_cd) at different temperatures^5–7, 12^, which evolve from a high-temperature *β* ($\mathrm{Fm}\bar{{\rm{3}}}m$) phase^6, 8, 12^. The *β* phase belongs to an antifluorite structure in which an arsenic atom is coordinated by six cationic and two vacancies randomly distributed in corners of a cube. In the low-temperature phases, two cationic atoms are missed along a diagonal of one face in the distorted cube^5–7, 12^. At room temperature, both Cd_3_As_2_ and Zn_3_As_2_ were reported to crystallize in a body-center tetragonal phase^6, 7, 12, 19–26^. Recently single-crystal XRD measurements suggested that the crystals of Cd_3_As_2_ form in the structure of I4_1_/acd instead of I4_1_/cd at room temperature^9^. Considering that our powder XRD cannot distinguish these two structures, we prefer to believe the result in ref. 9 and take I4_1_/acd to be *α*-phase hereafter. The unit cell of I4_1_/acd phase is made of the unit cells of P4_2_/nmc phase associated with the lattice constants ${{\rm{a}}}_{{\rm{I}}}=\sqrt{2}{{\rm{a}}}_{{\rm{P}}}$ and c_I_ = 2c_P_ (The subscripts I and P present the body-center and primitive space group respectively) (Fig. 1(a)-(d)).

**Figure 2:**
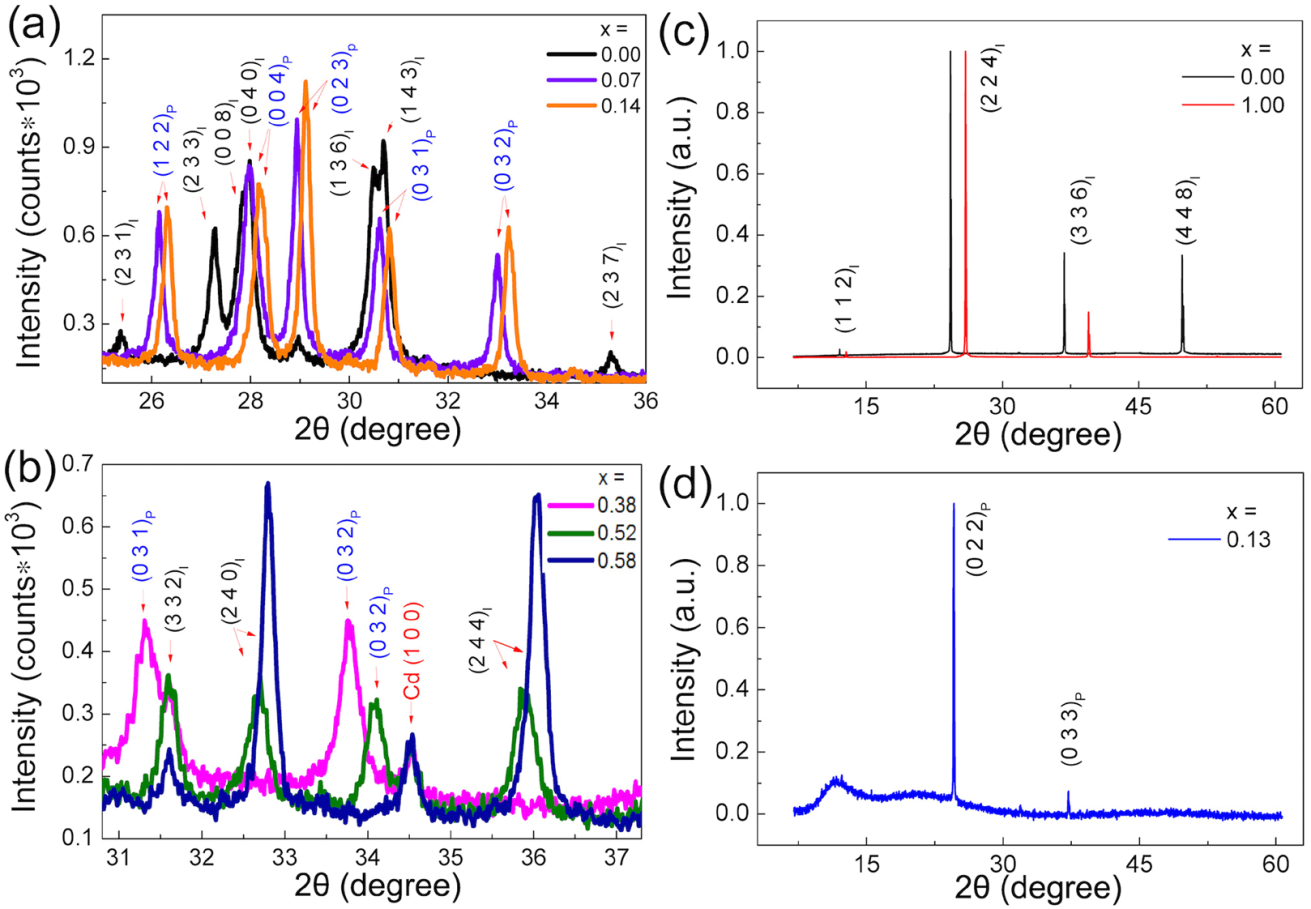
XRD patterns for (Cd_1−x_Zn_x_)_3_As_2_. Panel (a): Powder XRD patterns for x = 0, 0.07 and 0.14 for 25° < 2*θ* < 36°. The characteristic peaks of (231)_I_, (233)_I_ and (237)_I_ for a body-center structure and (032)_P_ for a primary structure are labeled. Panel (b): Powder XRD patterns for x = 0.38, 0.52 and 0.58 for 31° < 2θ < 37°. The peak of (032)_P_ occurs for x = 0.38, while the peaks of (240)_I_ and (244)_I_ occur for x = 0.58. For x = 0.52, we observed two sets of peaks, which indicates the batch has the mixture of two types of crystals. The (100) peak of cadmium at 34.5° occurs in all the XRD patterns with no shift. Panel (c): The diffraction pattern of the (112)_I_ plane for α − Cd_3_As_2_ and α − Zn_3_As_2_. Panel (d): The diffraction pattern of the (022)_P_ plane for (Cd_0.87_Zn_0.13_)_3_As_2_.

Our powder XRD measurements revealed that (Cd_1−x_Zn_x_)_3_As_2_ have different crystal structures for different x at room temperature. Figure 2(a) shows that the crystals of Cd_3_As_2_ grown from flux are *α* phase, and their XRD pattern exhibits the characteristic peaks of (231)_I_, (233)_I_ and (237)_I_ of the body-center tetragonal phase for 25° < 2*θ* < 36°. This result is consistent with what was previously reported^7, 19^. Once a small amount of Zn is added (x = 0.07), the XRD pattern is distinct from that of Cd_3_As_2_. The characteristic peaks of (231)_I_, (233)_I_ and (237)_I_ disappear, while the (032)_P_ peak of the P4_2_/nmc group occurs (Fig. 2(a)). This peak remains resolvable until the doping level reaches x = 0.38. For 0.38 < x < 0.46, the structure reenters the body center tetragonal structure I4_1_/acd accompanied by (240)_I_ and (244)_I_ peaks which are exceedingly weak in the pattern of Cd_3_As_2_^7, 36, 37^ (Fig. 2(b)).

**Figure 3:**
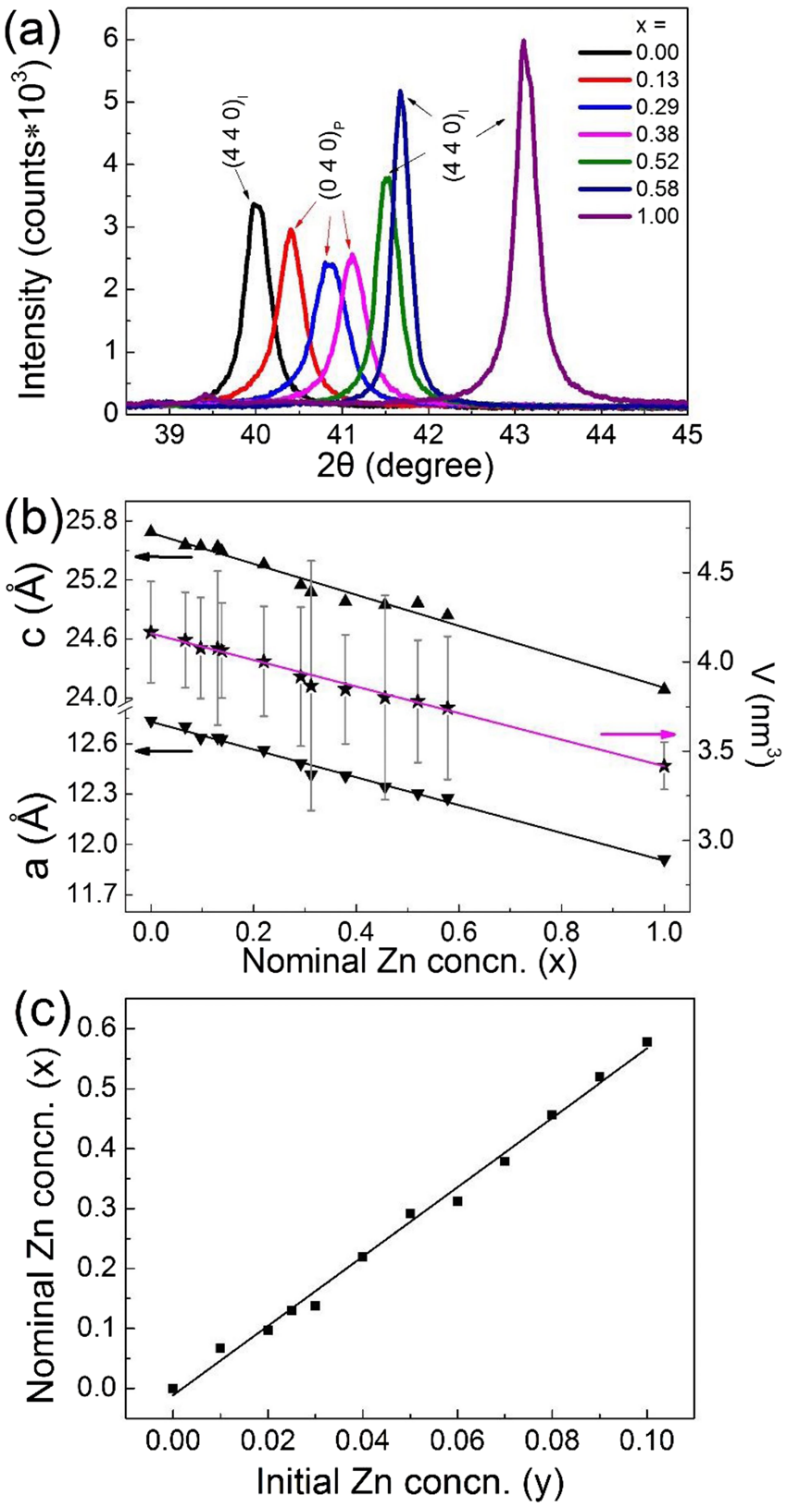
Panel (a): The (440)_I_ peaks and the counterpart (040)_P_ peaks in the powder XRD patterns for (Cd_1−x_Zn_x_)_3_As_2_. Panel (b): The lattice constants and the volume of the primitive cell change with the concentration (concn.) of zinc x linearly. Panel (c): The nominal concentration of zinc x and the initial y have a linear dependence for 0.00 ≤ x < 0.58.

The peaks of (440)_I_ with the strongest intensity stand at 40.0° and 43.1° for α − Cd_3_As_2_ and Zn_3_As_2_ respectively (Fig. 3(a)). The peak of (040)_P_ is the counterpart of (440)_I_ in P4_2_/nmc group. Figure 3(a) shows that the peaks of (040)_P_ and (440)_I_ shift gradually when x changes from 0 to 1. Although the volume of the unit cell changes about 20% from x = 0 to 1, the peak shape does not change significantly in the solid solutions, indicating homogeneous chemical distributions in the crystals. The lattice constants for the samples in the I4_1_/acd group were presented in the view of the P4_2_/nmc group as ${{\rm{a}}}_{{\rm{I}}}=\sqrt{2}{{\rm{a}}}_{{\rm{P}}}$ and c_I_ = 2c_P_. Figure 3(b) shows that a_P_ and c_P_ change in a precisely linear relation with respect to x, albeit the structural transitions. This result is similar as what is reported for polycrystalline (Cd_1−x_Zn_x_)_3_As_2_^36^.

**Figure 4:**
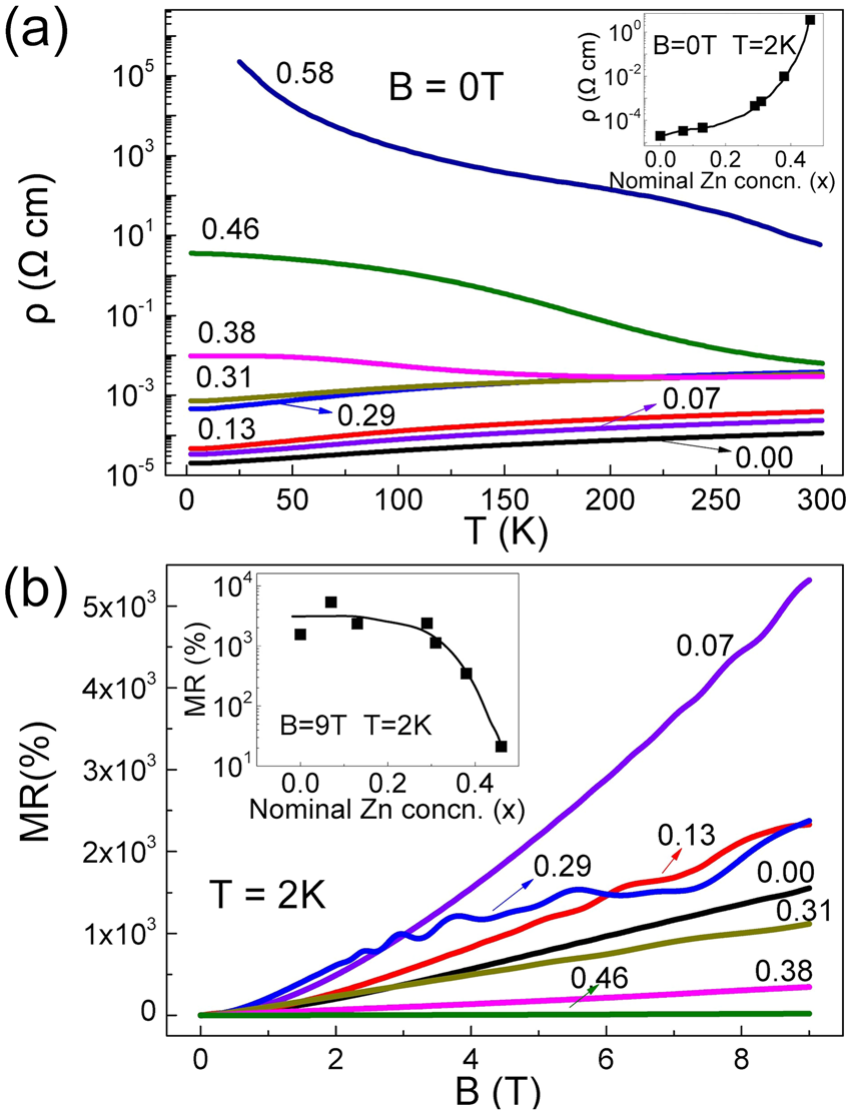
Panel (a): Temperature dependent resistivity of (Cd_1−x_Zn_x_)_3_As_2_ for 0 ≤ x ≤ 0.58 in zero magnetic field. Inset: The values of resistivity for 0 ≤ x ≤ 0.46 at 2 K in zero magnetic field. Panel (b): MR versus magnetic fields at 2 K. Inset: The values of MR for (Cd_1−x_Zn_x_)_3_As_2_ (0 ≤ x ≤ 0.58) versus x at 9 T at 2 K.

Temperature dependent resistivity of (Cd_1−x_Zn_x_)_3_As_2_ shows a clear change from a metallic to semiconducting profile when x increases from 0~0.31 to 0.38~0.58 (Fig. 4(a)). The resistivity of Cd_3_As_2_ is close to what was reported in ref. 22 with the residual resistivity ratio (RRR = *ρ*_(T=300K)_/*ρ*_(T=2K)_) being 10. The values of RRR keep almost invariant when x increases up to 0.31. For x = 0.38, the *ρ*(T) decreases with decreasing temperatures above 200 K, and then increases below this temperature. This complicated behavior indicates that the sample is likely a very narrow bandgap semiconductor for x = 0.38. The values of RRR then dramatically decrease for x ≥ 0.38, being 0.58 for x = 0.38 and 7.7 × 10^−6^ for x = 0.58 (see more details in the final phase diagram). The changes of the RRR for different x indicate a process of band gap opening for x ≥ 0.38. Such metal-semiconductor transition point is close to that reported for polycrystals^36^.

Figure 4(b) shows the MR for the samples for x ≤ 0.46 at 2 K. When x ≤ 0.31, the values of the MR are comparably large as that for Cd_3_As_2_^25, 38^. The values of the MR decline significantly for the semiconducting samples for x ≥ 0.38. Recent studies of Cd_3_As_2_ reported linear MR at low temperatures^25, 26^. In our experiments, the MR of (Cd_1−x_Zn_x_)_3_As_2_ follows the power law of MR ∝ H^α^ where α varies from 0.9 to 1.5 for different samples.

**Figure 5:**
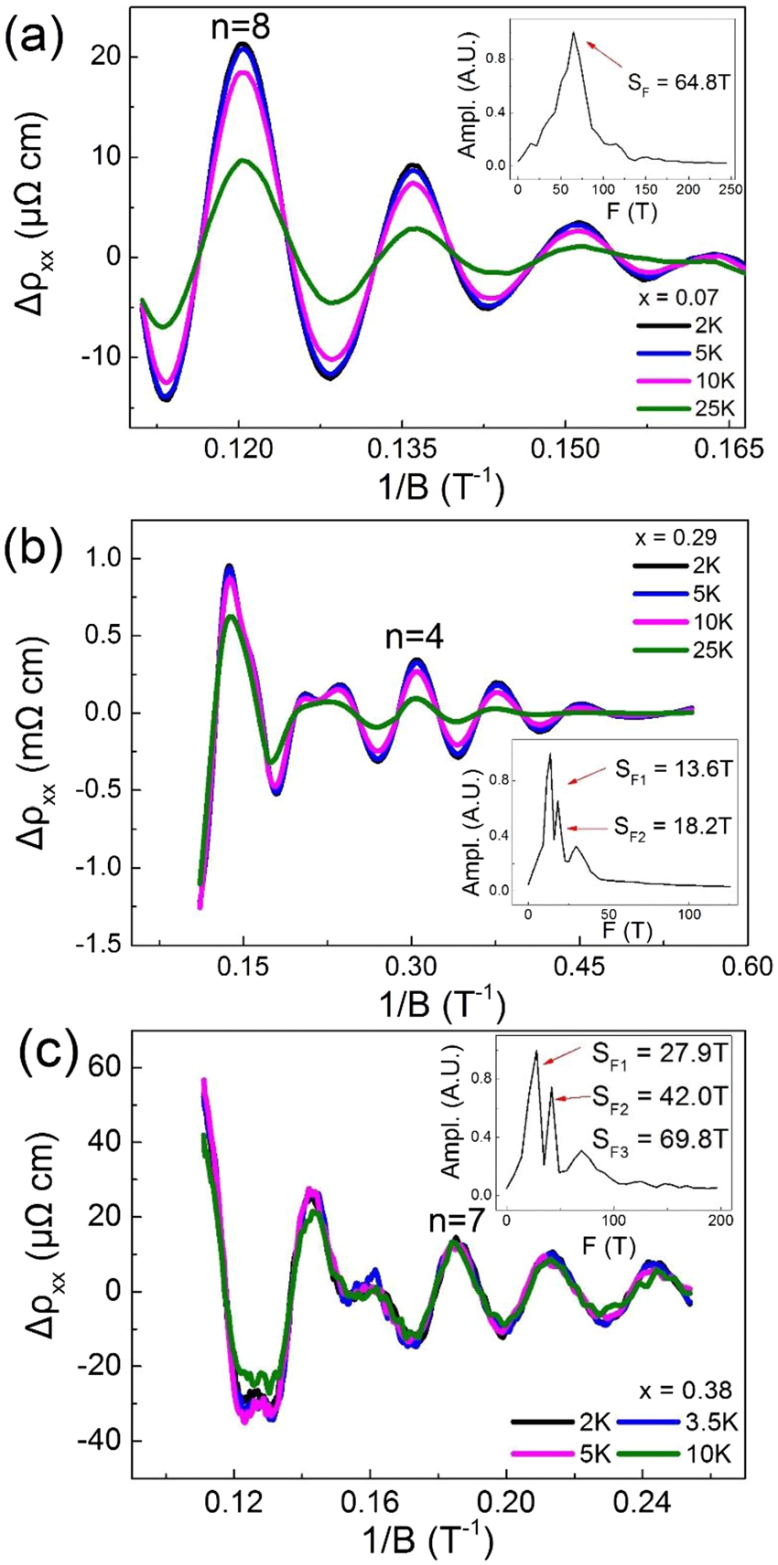
The oscillatory components of Δ*ρ*_xx_ versus the reciprocal of the magnetic field (1/B) at different temperatures for x = 0.07, 0.29 and 0.38. Insets: Fast Fourier Transform (FFT) spectra for x = 0.07, 0.29 and 0.38. The results of the SdH oscillations are summarized in Table 1.

**Figure 6:**
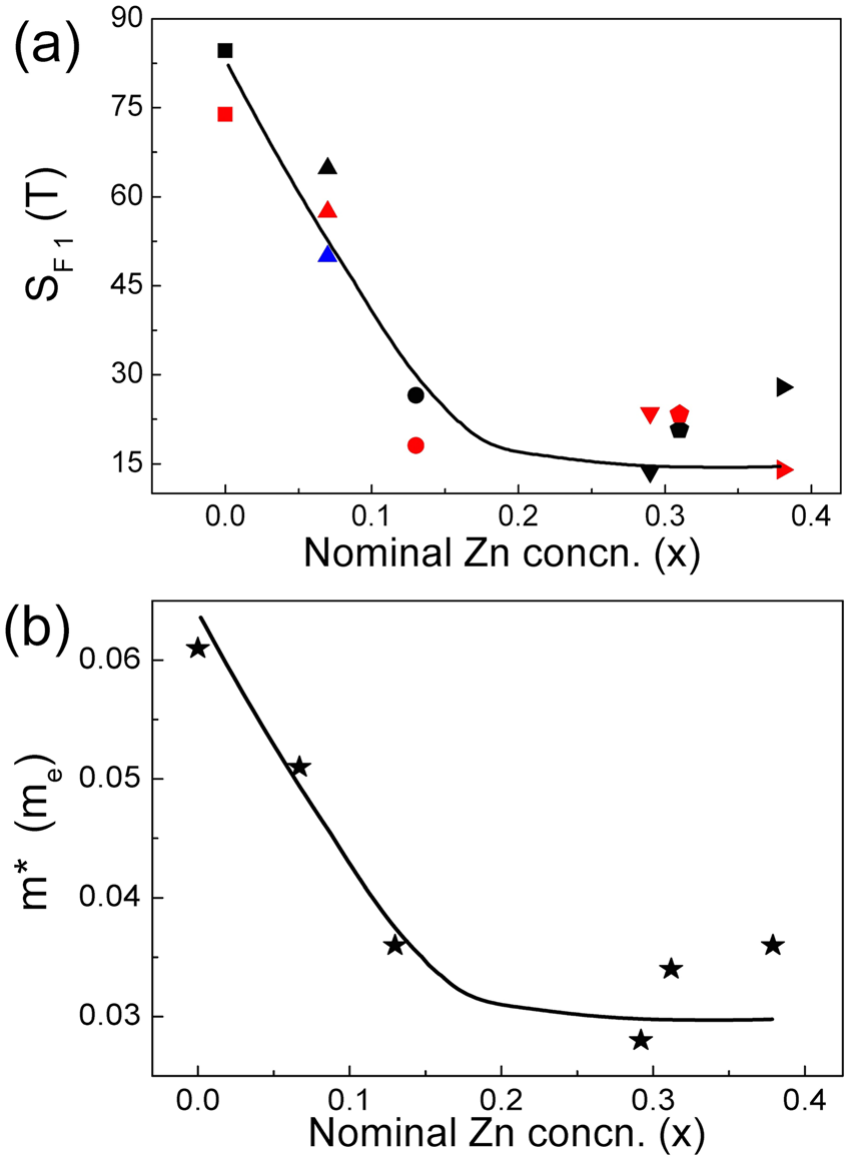
Panel (a): The main frequency of the SdH oscillations at 2 K for (Cd_1−x_Zn_x_)_3_As_2_. Different colors represent different samples from the same batches. The black ones are the samples corresponding to Figs 4(a) and 7(b). Panel (b): The cyclotron effective mass m^*^ according to the main frequency for each sample versus x. The line is for visual guidance.

Here we observed the precious studies reported the SdH oscillations for (Cd_1−x_Zn_x_)_3_As_2_ when x ≤ 0.1 and x = 0.2^39, 40^. Conspicuous SdH oscillations occur in the field dependent resistivity for all the samples for x ≤ 0.38 here at low temperatures, while the oscillations were not observed for the samples for x ≥ 0.46. This observation is consistent with a metal-insulator transition with a critical point x_c_ ~ 0.38. The part of resistivity with oscillations versus the reciprocal of the magnetic field is presented in Fig. 5. For x < 0.29, only one frequency was observed for each sample (Fig. 6(a)). The frequencies of the oscillations show a clear trend of a decline with respect to x up to 0.29 (Fig. 6(a)). For 0.29 ≤ x ≤ 0.38, the frequencies show more significant sample difference in a same batch. Some samples show single frequencies from 15 T to 30 T, while the second and third frequencies as large as 70 T occur in other samples. Such strong sample-dependence and complicated multi-frequency features indicate that the samples for 0.29 ≤ x ≤ 0.38 are semimetals or very narrow bandgap semiconductors with complicated Fermi surface which is strongly influenced by subtle changes of chemical potential.

As shown in Fig. 5, the temperature dependent amplitudes of the SdH oscillations were fitted by the Lifshitz-Kosevich formula^41–44^:

1 $${\rm{\Delta }}{\rho }_{{\rm{xx}}}\propto {A(T)e}^{-\frac{{\rm{2}}{\pi }^{{\rm{2}}}{{\rm{k}}}_{{\rm{B}}}{{\rm{T}}}_{{\rm{D}}}}{\hslash {\omega }_{{\rm{c}}}}}\,\cos \,{\rm{2}}\pi (\frac{{{\rm{S}}}_{{\rm{F}}}}{{\rm{B}}}+\beta )$$

2 $$A(T)=\frac{{\rm{2}}{\pi }^{{\rm{2}}}{{\rm{k}}}_{{\rm{B}}}{\rm{T}}/\hslash {\omega }_{{\rm{c}}}}{\sinh ({\rm{2}}{\pi }^{{\rm{2}}}{{\rm{k}}}_{{\rm{B}}}T/\hslash {\omega }_{{\rm{c}}})}$$

where k_B_ is the Boltzmann’s constant; ω_c_ is the cyclotron frequency; T_D_ is the Dingle temperature and A(T) is the thermal damping factor which helps to fit the energy gap $\hslash$ω_c_. For the samples with multi-frequencies, their main frequencies were analyzed. For large x, the amplitudes of the oscillations are damped less significantly by the temperatures, which indicates a smaller cyclotron effective mass (Fig. 5).

The parameters of the SdH oscillations for different x are listed in Table 1. The cross-sectional area A_F_ in the momentum space comes from the Onsager relation ${{\rm{S}}}_{{\rm{F}}}=\frac{\hslash }{2{\rm{\pi }}e}{{\rm{A}}}_{{\rm{F}}}$. Simply assuming a circular A_F_, we got the Fermi wave vector k_F_ from ${{\rm{A}}}_{{\rm{F}}}={{\rm{\pi }}k}_{{\rm{F}}}^{{\rm{2}}}$. The Fermi velocity ν_F_ = $\hslash$k_F_/m^*^, the Fermi energy ${{\rm{E}}}_{{\rm{F}}}={{\rm{\nu }}}_{{\rm{F}}}^{{\rm{2}}}{{\rm{m}}}^{\ast }$, and the cyclotron effective mass m^*^ = eB/ω_c_ are listed as well. Figure 6 shows that m^*^ changes in a similar manner as S_F1_ with respect to x.

**Table 1:** Parameters of the tested samples of different zinc concentration x.

x	0.00	0.07	0.13	0.29	0.31	0.38
Parameters						
A_F_(10^−3^ Å^−2^)	8.08	6.18	2.53	1.30/1.74	1.98	2.66/4.01/6.66
k_F_(Å^−1^)	0.051	0.044	0.028	0.02/0.024	0.025	0.029/0.036/0.046
E_F_(eV)	0.346	0.289	0.165	0.107/−	0.143	0.169/−/−
ν_F_(10^5^ m/s)	9.9	10.1	9.1	8.3/−	8.6	9.4/−/−
m^*^(m_e_)	0.061	0.051	0.036	0.028	0.034	0.036

The dashed entries mean quantities missing.

**Figure 7:**
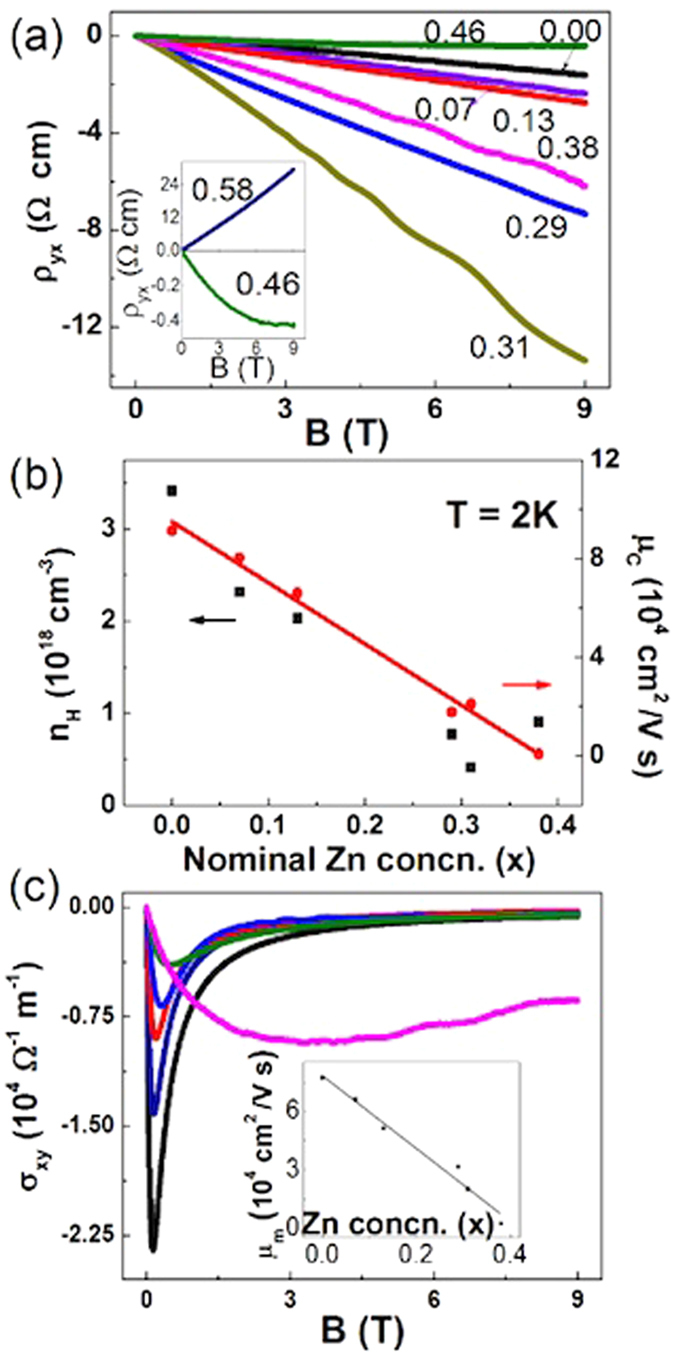
Panel (a): Field dependent Hall resistivity of (Cd_1−x_Zn_x_)_3_As_2_ for 0 ≤ x ≤ 0.46 at 2 K. From x = 0.00 to 0.46, all samples are n-type. Inset: The Hall resistivity of the sample for x = 0.46 shows a non-linear profile. For x = 0.58, the signal turns to p-type. Panel (b): Carrier density n_H_ (n_H_ = B/(e*ρ*_yx_)) and mobility *μ*_c_(*μ*_c_ = 1/(e*ρ*_xx_n_H_)) at 2 K versus x for x = 0.00 to 0.38. Panel (c): The conductivity σ_xy_ versus the magnetic field. Inset: The mobility got from the standard Bloch-Boltamann transport.

In order to better understand the metal-insulator transition, we measured Hall resistivity in (Cd_1−x_Zn_x_)_3_As_2_ at 2 K. The field dependent Hall resistivity of the samples for x ≤ 0.31 shows a linear negative profile with SdH oscillations on the background. The negative linear-field-dependent ρ_yx_(H) indicates that the carriers in the samples for x ≤ 0.31 simply originate from an electron band. The carrier density decreases nearly linearly with increasing x from 0 to 0.31 (Fig. 7(b)). These results are consistent with the observation of the decreasing SdH oscillation frequencies with respect to x^36, 39, 40^. ρ_yx_(H) becomes smaller and nonlinear for 0.38 ≤ x < 0.59. This nonlinear feature is clear for x = 0.46 (inset of Fig. 7(a)). In this range, the samples manifest a semiconducting ρ(T) profile while multi-frequencies were observed in the SdH oscillations in their MR. The Hall signals and the resistivity indicate two types of carriers. For x ≥ 0.59, the Hall signals become large and positively field-dependent, which indicate p-type semiconductors consistent with low carrier concentrations. The change of the carrier density n_H_ and mobility μ_c_ with respect to x is summarized in Fig. 7(b,c). Meanwhile we used the standard Bloch-Boltzmann transport to estimate the mobilities which read μ_m_ = 1/B_max_ as the minimum on the σ_xy_ curves in Fig. 7(c). The mobility undergoes a similar linear decline with the respect to x.

## Discussion

Our measurements show that the change of the electrical properties of (Cd_1−x_Zn_x_)_3_As_2_ has no observable correlation with the structural transitions. This result is not unexpected according to previous band structural calculation. Both α′′ (P4_2_/nmc) and α (I4_1_/acd) phases of Cd_3_As_2_ manifest similar simple band structures near the Fermi surface. Only two Dirac cones protected by rotational symmetry cross the Fermi level (E_F_) along the high symmetric line Γ − Z in the Brillouin zones^18^. Therefore a structural transition cannot influence the bands near the E_F_.

Previous studies of the calculation and experiments showed that the negative gap is about $-{\rm{0.3}}\,{\rm{eV}} \sim -{\rm{0.7}}\,{\rm{eV}}$ for Cd_3_As_2_^18, 35^, while the direct gap is 1.0 eV for Zn_3_As_2_^13^. With a semimetal and a semiconductor as two terminals, the band inversion transition should accompany a metal-semiconductor transition at a certain x. If we assume that the band gap of (Cd_1−x_Zn_x_)_3_As_2_ changes linearly with respect to x^35^, the critical point of the band inversion transition is estimated to occur in the range of 0.23 ≤ x ≤ 0.41. This estimation is consistent with our experimental results. The critical point can also be estimated by considering the change of the SOC strength in (Cd_1−x_Zn_x_)_3_As_2_. Here we assume that the band inversion is solely induced by the change of the SOC strength, which is proportional to Z^4^/n^3^ in case of the hydrogenic wavefunctions in a Coulomb field where Z is the nuclear charge and n is the principal quantum number^45^. Then the critical point is estimated as ${\rm{x}} \sim {\rm{0.35}}$, which is very close to the experimental result: ${{\rm{x}}}_{{\rm{c}}} \sim {\rm{0.38}}$.

The band structure calculation for Cd_3_(As_1−x_P_x_)_2_ revealed a topological phase transition from a Dirac semimetal to a trivial semiconductor induced by the change of the SOC strength^31^. When x increases, the two Dirac points along the k_z_ axis gradually move closer and then merge at the Γ point under the protection of the crystal symmetry. A direct band gap is opened beyond the critical point. The process of the band inversion transition in (Cd_1−x_Zn_x_)_3_As_2_ should be similar as that in Cd_3_(As_1−x_P_x_)_2_. Despite of large chemical replacement in the crystals, the Dirac cones are robust, which is supported by the vanishing disorder self-energy around the crossing points^46^. Further investigation such as ARPES measurements for (Cd_1−x_Zn_x_)_3_As_2_ will help to reveal the details of this topological phase transition.

Cd_3_As_2_ is always n-type due to As vacancies, while Zn_3_As_2_ is p-type because extra Zn vacancies serve as electron acceptors^13, 38^. Since both two types of carriers come from element vacancies, an n to p transition is expected in (Cd_1−x_Zn_x_)_3_As_2_. With increasing x, the zinc doping will suppress the chemical potential, which crosses a small Fermi surface near the Dirac cones. The decrease of S_F1_ with increasing x is a comprehensive result of the change of the band structure and chemical potential.

For 0.29 ≤ x ≤ 0.38, we found strongly sample-dependent frequencies of SdH oscillations. For a very narrow bandgap semiconductor or semimetal, any small change of the carrier concentrations will affect the chemical potential dramatically near the band touching. The strong sample-dependence and the complicated SdH oscillations in this regime are not unexpected.

### Summary

**Figure 8:**
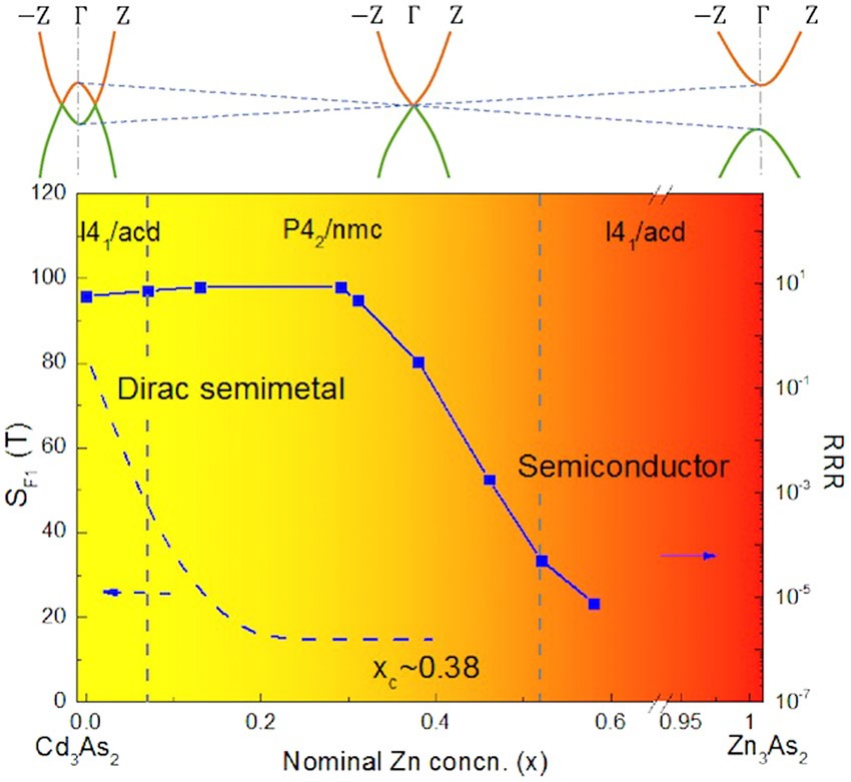
Phase diagram for (Cd_1−x_Zn_x_)_3_As_2_. With the concentration of Zn increasing, the samples transform from a topological Dirac semimetal to a semiconductor. The upper sketches of the band structure illustrate this transition. The structure transforms from I4_1_/acd to P4_2_/nmc then back to I4_1_/acd with vertical dashed lines serving as rough boundaries. The background color presents the gradual change of resistivity at 2 K as x increases (inset of Fig. 4(a)). The changes of S_F1_ and RRR are plotted in the diagram as well.

Single crystals of (Cd_1−x_Zn_x_)_3_As_2_ were synthesized from high-temperature solutions. Based on the analysis of the electrical properties, we realized a transition from a 3D Dirac semimetal to a semiconductor with the critical point ${{\rm{x}}}_{{\rm{c}}} \sim {\rm{0.38}}$ in these solid solutions (Fig. 8). The structural transitions do not affect the electrical properties in this system. The topological aspect of this metal-insulator transition needs experimental exploration in the future.

## Methods

Single crystalline (Cd_1−x_Zn_x_)_3_As_2_ samples were grown from high temperature solutions with the initial concentration of starting elements being (Cd_1−y_Zn_y_)_9_As_1_. The mixtures were sealed in evacuated quartz ampoules, and then kept at a high temperature between 800 °C and 1100 °C for two days, and then slowly cooled down to 425 °C with a rate of −5 °C/hour. After staying at 425 °C for one day, the ampoules were centrifuged to separate crystals from flux. The single crystals of Cd_3_As_2_ were mainly 3D bulks with triangular facets, but several needle-like crystals were found in the growth as well. The shapes of the crystals were similar as that described in refs 20, 21. When zinc was added to the solutions, the sizes of the 3D crystals decreased, while some flake-like crystals with smooth or mesa-landscape-like surfaces appeared. For x ≥ 0.58, the flake-like crystals were dominant and no 3D crystals appeared in the growth. XRD measurements revealed that both the triangular facets (Fig. 1(f)) and the large surface of the flake-like crystals (Fig. 1(e)) were either the (011)_P_ face of the P4_2_/nmc structure or the (112)_I_ face of the I4_1_/acd structure, which were the counterparts of each other (Fig. 2(c,d)). In a same batch of growth, the crystals with different morphologies did not show larger difference of physical properties than those with the same morphologies.

In order to determine the zinc concentration x, we measured the Energy Dispersive X-ray Spectrum (EDX) of the samples in an FEI Nova NanoSEM 430 spectrometer. The samples with no residual cadmium were selected in the measurements and their EDX spectrum was observed through an overall area scanning. The linear relation of the measured zinc concentrations x and the initial y (0 ≤ y ≤ 0.1) is shown in Fig. 3(c). By weighing the mass of the crystals yielded in every growth, we found that all the initial stoichiometric zinc was compounded in the crystals.

The powder XRD data was collected from a Rigaku MiniFlex 600 diffractometer and then refined by a Rietica Rietveld program. As shown in Fig. 3(b), the lattice constants and the volume of the unit cell change linearly with x in accordance with a Vegards law. This result is same with what observed in polycrystalline (Cd_1−x_Zn_x_)_3_As_2_^36, 47^. Therefore we selected x determined by EDX measurements as the nominal zinc concentrations, which were estimated to have less than ± 1% difference between the real zinc concentrations. More details of the XRD experiments are discussed in the Result part.

Single crystals were polished to the bars with length ~1.0 mm, width ~0.4 mm and thickness ~0.3 mm for electrical transport measurements. The crystallographic orientation of the bars was same as those chosen in the recent experiments^21–25^, i.e., the current was perpendicular to the [112]_I_ direction in I4_1_/acd (or [011]_P_ direction in P4_2_/nmc), and the magnetic field was along the [112]_I_ direction in I4_1_/acd (or [011]_P_ direction in P4_2_/nmc). The electrical resistance and Hall voltage were measured via a four-point contact method in Quantum Design Physical Property Measurement System (PPMS-9).

## References

[1] Żdanowicz W, Żdanowicz L (1975). Semiconducting compounds of the A^II^B^V^ group. Annu. Rev. Matter. Sci..

[2] Misiewicz J (1994). Zn_3_P_2_—a new material for optoelectronic devices. Microelectronics J.

[3] Stepanchikov D, Shutov S (2006). Cadmium phosphide as a new material for infrared converters. Sem. Phys. Quant. El. & Opt..

[4] Burgess T (2015). Zn_3_As_2_ nanowires and nanoplatelets: highly efficient infrared emission and photodetection by an earth abundant material. Nano Lett..

[5] Stackelberg MV, Paulus R (1935). Investigation on phosphides and arsenides of zinc and cadmium. the Zn_3_P_2_ lattice. Z. Phys. Chem..

[6] Weglowski S, Łukaszewicz K (1968). Phase transition of Cd_3_As_2_ and Zn_3_As_2_. Bull. Acad. Polon. Sci. Ser. Sci. Chim.

[7] Steigmann GA, Goodyear J (1968). The crystal structure of Cd_3_As_2_. Acta Cryst. B.

[8] Pistorius CWFT (1975). Melting and polymorphism of Cd_3_As_2_ and Zn_3_As_2_ at high pressures. High Temp. High Press.

[9] Ali MN (2014). The crystal and electronic structures of Cd_3_As_2_, the three-dimensional electronic analogue of graphene. Inorg. Chem..

[10] Elrod U (1984). Morphological and structural properties of Zn_3_P_2_ single crystals grown by recrystallization in a closed system. J. Cryst. Growth.

[11] Pistorius CWFT, Clark GB, Ceotzer J, Kruger GJ, Kunze OA (1977). High pressure phase relations and crystal structure determination for Zn_3_P_2_ & Cd_3_P_2_. High Press. High Temp..

[12] Rubtsov VA, Trukhan VM, Yakimovich VN (1990). Thermal expansion of (Cd_1−x_Zn_x_)_3_(P_1−y_As_y_)_2_ solid solutions. Dokl. Akad. Nauk BSSR.

[13] Turner WJ, Fischler AS, Reese WE (1961). Physical properties of several 2–5 semiconductors. Phys. Rev..

[14] Fagen EA (1979). Optical properties of Zn_3_P_2_. J. Appl. Phys..

[15] Haacke G, Castellion GA (1964). Preparation and semiconducting properties of Cd_3_P_2_. J. Appl. Phys..

[16] Aubin MJ, Caron LG, Jay-Gerin JP (1977). Band structure of cadmium arsenide at room temperature. Phys. Rev. B.

[17] Caron LG, Jay-Gerin JP, Aubin MJ (1977). Energy-band structure of Cd_3_As_2_ at low temperatures and the dependence of the direct gap on temperature and pressure. Phys. Rev. B.

[18] Wang Z, Weng H, Wu Q, Dai X, Fang Z (2013). Three-dimensional *Dirac* semimetal and quantum transport in Cd_3_As_2_. Phys. Rev. B.

[19] Neupane M (2014). Observation of a three-dimensional topological *Dirac* semimetal phase in high-mobility Cd_3_As_2_. Nat. Commun..

[20] Liu ZK (2014). A stable three-dimensional topological *Dirac* semimetal Cd_3_As_2_. Nat. Mater..

[21] Borisenko S (2014). Experimental realization of a three-dimensional *Dirac* semimetal. Phys. Rev. Lett..

[22] Jeon S (2014). Landau quantization and quasiparticle interference in the three-dimensional *Dirac* semimetal Cd_3_As_2_. Nat. Mater..

[23] Liang T (2015). Ultrahigh mobility and giant magnetoresistance in the *Dirac* semimetal Cd_3_As_2_. Nat. Mater..

[24] Zhao Y (2015). Anisotropic *Fermi* surface and quantum limit transport in high mobility three-dimensional *Dirac* semimetal Cd_3_As_2_. Phys. Rev. X.

[25] He LP (2014). Quantum transport evidence for the three-dimensional *Dirac* semimetal phase in Cd_3_As_2_. Phys. Rev. Lett..

[26] Narayanan A (2015). Linear magnetoresistance caused by mobility fluctuations in *n*-doped Cd_3_As_2_. Phys. Rev. Lett..

[27] Orlita M (2014). Observation of three-dimensional massless *Kane* fermions in a zinc-blende crystal. Nat. Phys.

[28] Dziawa P (2012). Topological crystalline insulator states in Pb_1−x_Sn_x_Se. Nat. Mater..

[29] Xu S-Y (2011). Topological phase transition and texture inversion in a tunable topological insulator. Science.

[30] Brahlek M (2012). Topological-metal to band-insulator transition in (Bi_1−x_In_x_)_2_Se_3_ thin films. Phys. Rev. Lett..

[31] Narayan A, Di Sante D, Picozzi S, Sanvito S (2014). Topological tuning in three-dimensional *Dirac* semimetals. Phys. Rev. Lett..

[32] Żdanowicz W, Łukaszewicz K, Trzebiatowski W (1964). Crystal structure of semiconducting system Cd_3_As_2_-Zn_3_As_2_. Bull. Acad, Pol. Sci., Ser. Chim.

[33] Żdanowicz L, Żdanowicz W (1964). Semiconducting properties of (Cd_1−x_Zn_x_)_3_As_2_-type solid solutions. Phys. Stat. Sol..

[34] Rogers LM, Jenkins RM, Crocker AJ (1971). Transport and optical properties of the Cd_3−x_Zn_x_As_2_ alloy system. J. Phys. D: Appl. Phys..

[35] Wagner RJ, Palik ED, Swiggard EM (1971). Interband magnetoabsorption in Cd_x_Zn_3−x_As_2_ and Cd_3_As_x_P_2−x_. J. Phys. Chem. Solids, Suppl..

[36] Castellion GA, Beegle LC (1965). The preparation and properties of Cd_3_As_2_-Zn_3_As_2_ alloys. J. Phys. Chem. Solids.

[37] Volodina GF, Zakhvalinskii VS, Kravtsov VK (2013). Crystal structure of *α*′′′-(Zn_1−x_Cd_x_)_3_As_2_ (x = 0.26). Crystallogr. Rep..

[38] Iwami M, Matsunami H, Tanaka T (1971). Galvanomagnetic effects in single crystals of cadmium arsenide. J. Phys. Soc. Jan..

[39] Aubin MJ, Portal JC (1981). Shubnikov-de Haas oscillations in Cd_3−*x*_Zn_*x*_As_2_ alloys. Solid State Commun..

[40] Arushanov EK (1988). Cyclotron masses and g-factors of electrons in Cd_3−x_Zn_x_As_2_ solid solutions. Fiz. Tekh. Poluprovodn.

[41] Qu D-X, Hor YS, Xiong J, Cava RJ, Ong NP (2010). Quantum oscillations and *Hall* anomaly of surface states in the topological insulator Bi_2_Te_3_. Science.

[42] Shoenberg, D. In *Magnetic oscillations in metal* (Cambridge University Press, Cambridge, 2009).

[43] Murakawa H (2013). Detection of *Berry*’s phase in a bulk *Rashba* semiconductor. Science.

[44] Bell C (2013). Shubnikov-de *Hass* oscillations in the bulk *Rashba* semiconductor. BiTeI. Phys. Rev. B.

[45] Khomskii, D. Role of spin-orbit coupling. In *Transition Metal compounds*, chap. 3, 71–78 (Cambridge University Press, Cambridge, 2014).

[46] Di Sante, D., Barone, P., Plekhanov, E., Ciuchi, S. & Picozzi, S. Robustness of *Rashba* and *Dirac* fermions against strong disorder. *Sci*. *Rep*. **5** (2015). 10.1038/srep11285 PMC4650895 26067146

[47] Naake HJ, Belcher SC (1964). Solid solutions in the system Zn_3_As_2_-Cd_3_As_2_. J. Appl. Phys..

